# The Impact of Telemonitoring on Improving Glycemic and Metabolic Control in Previously Lost-to-Follow-Up Patients with Type 2 Diabetes Mellitus: A Single-Center Interventional Study in the United Arab Emirates

**DOI:** 10.1155/2022/6286574

**Published:** 2022-04-20

**Authors:** Muhammad Hamed Farooqi, Dima Kamal Abdelmannan, Manal Mubarak Al buflasa, Moataz Abdalla Abbas Hamed, Maxon Xavier, Tessa Joyce Santos Cadiz, Faisal Amir Nawaz

**Affiliations:** ^1^Dubai Diabetes Center, Dubai Health Authority, Dubai, UAE; ^2^Cognitive Healthcare International, Dubai, UAE

## Abstract

**Background:**

Telemonitoring (TM), mobile-phone technology for health, and bluetooth-enabled self-monitoring devices represent innovative solutions for proper glycemic control, compliance and monitoring, and access to providers.

**Objective:**

In this study, we evaluated the impact of TM devices on glycemic control and the compliance of 38 previously lost-to-follow-up (LTFU) patients with type 2 diabetes mellitus (T2DM).

**Methods:**

This was an interventional single-center study that randomly recruited LTFU patients from the Dubai Diabetes Center (DDC), UAE. After contact and recruitment by phone, patients had an initial visit at which they were provided with home-based TM devices. A follow-up visit was conducted three months later.

**Results:**

The mean HbA1c decreased significantly from 10.3 ± 1.9% at baseline to 7.4 ± 1.5% at the end of follow-up, with a mean difference (MD) of −2.9% [95% CI: −3.6 to −2.2]. The percentage of patients with HbA1c <7% was 50% after three months. Home-based blood sugar monitor devices showed a significant reduction in fasting blood glucose (FBG) after three months (MD = -40.1 mg/dL, 95% CI: −70.8 to −9.3). A significant reduction was observed in terms of body weight after three months (MD = −1.3 kg, 95% CI: −2.5 to −0.08). The mean number of days the participants used a device was the highest for portable pill dispensers (86.5 ± 22.8 days), followed by a OneTouch® blood glucose monitor (72.9 ± 23.5 days).

**Conclusions:**

TM led to significant improvements in overall diabetes outcomes, including glycemic control and body weight, indicating its effectiveness in a challenging population of T2DM patients who had previously been lost to follow-up.

## 1. Introduction

In the Middle East and North Africa (MENA) region, diabetes affects 55 million people, with a notably higher prevalence (12.8%) than the global average (nearly 9.3%) in 2019 [1]. After Saudi Arabia and Kuwait, the United Arab Emirates (UAE) has one of the highest diabetes burdens in the Middle East (16.3%) [1–3]. Although several well-established behavioral and therapeutic interventions exist for diabetes, patient outcomes are still poor, with a high incidence of diabetes-related complications [4, 5]. Diabetes-related complications can be prevented or delayed with intensive glucose control. Nevertheless, up to 60%–78.2% of adult patients with diabetes in the MENA region are inadequately controlled [6–9]. In UAE, a five-year retrospective study noted that only 37.7% of the population with diabetes in Dubai had HbA1c <7% [9]. Generally, inadequate home blood glucose (BG) monitoring, nonadherence with medications or recommended lifestyle behaviors (nutrition and exercise), suboptimal patient education about the disease, and limited access to health experts are all factors that may lead to suboptimal BG control [10, 11]. Loss-to-follow-up (LTFU) is one of the primary drivers of poor diabetes outcomes in well-resourced countries [12, 13]. Patients are more likely to achieve adequate glucose control if they attend their scheduled visits; nonetheless, data from the MENA region highlight that a considerable proportion of patients with diabetes do not follow the recommended appointment schedules with their physicians [14, 15].

Therefore, several researchers proposed the application of telemedicine, including telemonitoring (TM) and teleconsultation, to optimize and improve the management of patients with T2DM. The cumulative body of evidence highlights that the application of telemedicine results in committed patients, which may improve glycemic control and reduce the need for hospital admissions [16]. Telemedicine and mobile-phone technology for health (mHealth), along with Bluetooth-enabled self-monitoring devices, can be effective solutions for educational challenges, compliance and monitoring, and access to providers [17]. BG control could be enhanced safely by adjusting drugs based on home BG readings reported to clinicians remotely [18]. Telemedicine can also be an efficient way to monitor diabetes complications, particularly macrovascular problems and comorbidities (e.g., arterial hypertension) [17]. The high penetration of mobile phones in most countries enables health programs and providers to engage with large numbers of patients directly. This can allow for monitoring patient health outcomes and adherence to medication and treatment regimens at the national, city, and individual levels with TM devices connected through mobile phones [19].

The necessity for the application of telemedicine has been widely recognized following the emergence of the coronavirus disease 2019 (COVID-19) pandemic. People with diabetes are classified as a high-risk group for severe COVID-19 illness and are advised to maintain social distancing measures [20]. These measures have negatively impacted the access of patients to healthcare providers [21]. For a chronic disease such as diabetes that requires careful BG monitoring along with recurrent physician consultation, telemedicine can be a viable alternative for patients seeking medical guidance without physical attendance to the clinics and increasing their risk of COVID-19 infection. Telemedicine represents a valuable tool for remote patient consultation and early recognition of possible diabetes complications, signs of blood glucose dysregulation, and infection [22].

This study aimed to evaluate the impact of TM devices, including home BG and vital signs monitoring devices, on the glycemic control and the compliance of previously LTFU patients with type 2 diabetes mellitus (T2DM).

## 2. Methods

We confirm that none of the study's procedures violated the principles of the latest version of the Declaration of Helsinki [23] and applicable local laws. The central institutional review board (IRB) of Dubai Health Authority, Dubai, UAE, approved the study protocol (DSREC-09/2019-06).

### 2.1. Study Design and Patients

The present study was an interventional, single-center, prospective trial, which was conducted at the DDC, Dubai, UAE. We recruited adult patients (aged ≥18 years) with an established diagnosis of T2DM and HbA1c >8% at the time of the study's initiation. Only patients who had missed their appointments for more than one year before the study's initiation were included. Patients who were familiar with the use of technology (self or dedicated family member) and provided written informed consent were included. We excluded patients receiving care primarily outside DDC, pregnant women or women who planned to become pregnant within six months from the study's initiation, and patients participating in any other clinical trial.

The study' investigators retrospectively reviewed the Dubai Diabetes Center (DDC) databases to select patients with T2DM lost to follow-up at their clinics. The study nurse contacted these subjects via phone call to check for eligibility and willingness to participate in the study and undergo a screening visit. A follow-up visit was conducted after three months, at the end of the study period.

### 2.2. Data Collection and TM Devices

At the initial study visit, the following data were collected from all eligible patients: demographics, medical history, history of previous medications, current medications (dosages and frequencies), body weight, vital signs, spirometry measurements, glycemic parameters, hemoglobin level, lipid profile, renal function tests, and urine analysis. All patients were provided with TM devices for home use. These included a OneTouch Select Plus Flex® blood glucose monitor (LifeScan Inc, Malvern, PA USA), electronic sphygmomanometer (Cognitive Healthcare International [CHI], European approval, CE mark), heart rate monitor and pulse oximeter (CHI, European approval, CE mark), and portable pill dispenser (CHI, European approval, CE mark). All patients were also provided with a dedicated phone with data connectivity only, which had the CHI app preloaded. Standardized training was provided to the patient on the use of the phone and CHI app.

Data from the CHI app were collected automatically, and the required responses were communicated back to the patient by clinic staff via a dedicated laptop. If the dedicated staff was away from the laptop, the data were available through their CHI app. Similarly, the study's investigators had the data available on their CHI app. Patients were instructed to complete daily data entry in their CHI app for three months. Patients were contacted for reminders or advice, as needed, based on the readings received from all of their TM devices.

At the center, the following devices were used at the screening and follow-up visits: BAYER DCA to test HbA1c (Vantage Siemens Healthcare, Bayer Diagnostics), portable electrocardiography (ECG) machine (CHI, European approval, CE mark), pulmonary function testing spirometer (CHI, European approval, CE mark), blood testing analyzer (CHI, European approval, CE mark), portable urine analyzer (CHI, European approval, CE mark), and weighing scale (CHI, European approval, CE mark). The incidence of adverse events was recorded throughout the study period.

### 2.3. Study Outcomes

The primary outcome was to assess the mean change from baseline in the HbA1c level after three months of use of TM devices. Additional outcomes measured included the three-month changes in fasting blood glucose (FBG) and random blood glucose (RBG), body weight, blood pressure, pulse rate, oxygen saturation, spirometer measurements, hemoglobin level, lipid profile, renal function tests, urine analysis, and ECG.

### 2.4. Sample Size Calculation and Statistical Analysis

According to estimates, this study needed 32 patients to detect an effect size of 0.5% between the average HbA1c at the final visit and the baseline visit using the two-sided paired *t*-test with 80% power and a 5% significance level. The effect size of 0.5% lies within the effect sizes reported by many studies, such as Yu et al. [24].

Data analysis was performed using the Statistical Package for Social Sciences (SPSS), version 24. Frequencies and percentages summarized categorical variables. Continuous variables were summarized by means and standard deviations (SDs) or median and interquartile range (IQRs) after checking the assumption of normality using the Shapiro-Wilk test. Data were presented with their 95% confidence interval (CI) for the estimate of the parameter, where applicable. Comparing two means was done using the Student's *t*-test for paired data. Comparing two categorical variables was done using the McNemar chi-square test. Spearman correlation was used to test the association between the change in HbA1c and the number of days glucose monitoring devices were used. No multivariate analyses were done for this study due to the small sample size. In the case of missing data, the denominator was reported in the body of the table. All statistical tests were two-sided. *p* values <0.05 were considered significant.

## 3. Results

### 3.1. Demographic and Clinical Characteristics

A total of 38 patients were included with a mean age of 48.2 ± 10.1 years. Patients were predominately female (57.9%). All participants had underlying conditions other than diabetes, mainly vitamin D deficiency (94.7%), dyslipidemia (89.5%), obesity (71.1%), hypertension (60.5%), and chronic kidney disease (36.8%). Sixty-five percent of the patients had microalbuminuria, and 35.7% had proteinuria. Additionally, coronary artery diseases and neuropathy were recorded in 13.2% and 10.5% of the patients, respectively (Table 1).

Most of the patients were on multiple medications. The median number of prior antidiabetic medications taken since the patient was first diagnosed with diabetes was 2 (3). Biguanides were among the top prescribed medications in 60.5% of the population, followed by dipeptidyl peptidase-4 (DPP4) inhibitors (55.3%) and insulin secretagogues (39.5%). Sodium-glucose cotransporter-2 (SGLT2) inhibitors were used in 28.9% of the patients. In terms of current medications, the median number of antidiabetic medications at the time of entry into the study was 4 (3), with biguanides (94.7%) and SGLT2 inhibitors (89.5%) accounting for the majority of current medications (Table 1). A detailed map of each patient's prior and current medications is presented in Supplementary material 1.

### 3.2. The Changes in Glycemic Parameters

**Figure 1:**
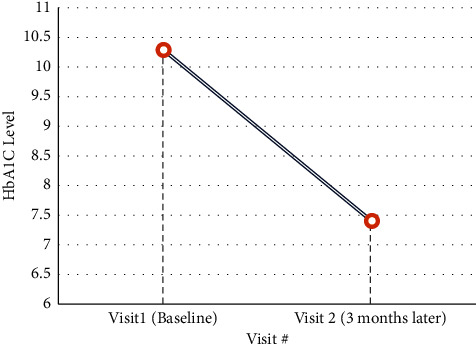
Mean HbA1C level at baseline and after three months measured in the center.

The mean HbA1c decreased significantly from 10.3 ± 1.9% at baseline to 7.4 ± 1.5% at the end of the third month of follow-up, with a mean difference (MD) of −2.9% [95% CI: −3.6 to −2.2, *p* < 0.001] (Figure 1). Overall, half of the patients (*n* = 19) achieved a HbA1c level of <7% after three months.

The average paired FBG exhibited a significant reduction from the baseline to the end of follow-up (MD = −40.1 mg/dL, 95% CI: −70.8 to −9.3, *p*=0.013) when measured using home-based BG monitors (Table 2). The same finding was observed using the center-based lab testing (Table 3). On the other hand, the average paired RBG did not change significantly from the baseline to the end of follow-up (MD = −40.8 mg/dL, 95% CI: −84.7 to 3.2, *p*=0.067) (Tables 2 and 3).

### 3.3. Changes in Other Parameters

The home-based measurements revealed no significant changes in systolic blood pressure, pulse rate, and oxygen saturation across the three months of follow-up. On the other hand, the diastolic blood pressure significantly decreased at the end of follow-up (−3.5 mmHg, 95% CI −6.6 to −0.4, *p*=0.028) (Table 2).

The center-based measurements of body composition markers, including weight, fat percent, muscle percent, water percent, and bone weight, showed that only weight exhibited a significant reduction at the end of follow-up (MD = −1.3 kg, 95% CI: −2.5 to −0.08, *p*=0.037). Analysis showed nonsignificant changes between baseline and measures recorded at the 3-month visit in terms of forced vital capacity (FVC; *p*=0.768), forced expiratory volume in one second (FEV1; *p*=0.947), peak expiratory flow (PEF; *p*=0.727), FEV1/FVC ratio (FEV1%; *p*=0.821), 25% flow of the FVC (FEF25; *p*=0.907), 75% flow of the FVC (FEF75; *p*=0.313), and average flow between 25% and 75% of the FVC (FEF25-75; *p*=0.683) (Table 3).

Laboratory testing showed a significant reduction in total cholesterol (MD = −20.6 mg/dL, 95% CI: −33.9 to −7.3, *p*=0.003) and low-density lipoprotein cholesterol (LDL-C; MD = −18.4 mg/dL, 95% CI: −29.5 to −7.3 *p*=0.002) after three months, compared to baseline measures. Other blood tests did not show significant changes at the end of follow-up. Likewise, none of the urine analysis markers showed significant changes at the end of follow-up, except for specific gravity, which decreased significantly (MD = −0.004, 95% CI: −0.008 to −0.0005, *p*=0.027). All ECG measurements were normal at baseline and after three months (Table 3).

### 3.4. The Usability of Home-Based TM Devices

The mean number of days the participants used a device was the highest for portable pill dispensers, with a mean of 86.5 ± 22.8 days. The OneTouch® Select Plus Flex® BGM was the second most used device with a mean of 72.9 ± 23.5 days. The electronic sphygmomanometer was used for a mean of 62.3 ± 28.6 days, while the pulse oximeter was used for 50.4 ± 28.6 days (Table 4). The mean number of reminders per patient was 2952 ± 935.5. The Spearman correlation showed a weak negative association between the frequency of BG monitor use and change in HbA1c (*r* = −0.028, *p*=0.866).

### 3.5. Safety Outcomes

No adverse events were reported by the participants.

## 4. Discussion

The current international guidelines recommend routine consultations every three months for patients with T2DM, particularly for poorly controlled patients [25]. Nonetheless, many patients were reported to skip regular face-to-face consultations. The traditional consultation method is relatively time-consuming for health care professionals and patients and ineffectively supports patient self-management [26]. TM, where the patient measures their signs and symptoms at home and makes them electronically available to their healthcare provider, is an intervention requiring input from patients and providers [27]. Many countries have used various TM strategies to manage T2DM, depending on their clinical circumstances [28]. Recent reports from the MENA region highlighted raised awareness about the benefits of TM among the general population and their willingness to use it [29]. To the best of our knowledge, this is the first study in the UAE that assessed the impact of TM on the management of patients with diabetes.

TM interventions, given via cellular phones and the Internet, have demonstrated their usefulness in multiple clinical trials in enhancing diabetes outcomes and lowering diabetes care costs [30]. Previous reports have shown that BG and blood pressure monitoring via TM of patients with T2DM was feasible and supported self-care and medical treatment decisions [31]. Nevertheless, health outcomes with diabetes TM systems have been varied, and a TM system by itself is unlikely to enhance outcomes [17]. The impact of TM systems varies depending on the level of patient engagement in diabetes self-management [32]. In the present study, we utilized a combination of various home- and clinic-based TM devices to ensure adequate monitoring of patients' statuses and prompt timely consultations and advice based on the readings received from these devices. Our findings demonstrated that TM was associated with a significant improvement in glycemic control after three months from the implementation of the TM system. Half of the patients achieved the targeted glycemic control at the end of follow-up.

Such findings are in good agreement with recent reports from different parts of the world. In the meta-analysis of Kim and his colleagues, a pooled analysis of 38 studies showed that TM was associated with a significant reduction in HbA1c levels of 6855 patients compared to usual care. This reduction was observed in the studies that monitored medication compliance, counseling, and education. In addition, they have demonstrated that the rate of achieving HbA1c <7% in the TM group was higher than usual care [33]. Lee et al. [34] conducted a randomized controlled trial that showed that TM (MyGlucoHealth, web-enabled glucometer) significantly reduced the HbA1c in a cohort of the population by a mean of 1.07% compared to only 0.24% in the usual care group (*p* < 0.01). Interestingly, they highlighted that compared with usual care, receiving TM was associated with a lower number of hypoglycemic events during Ramadan fasting and at the end of the study. Jeong et al. [35] reported a significant reduction in HbA1c among patients receiving TM devices for 24 weeks. The rate of patients who achieved HbA1c <7% was 33.9%. These findings suggest that TM could be used to encourage patients to acquire healthier habits. Thus, TM for diabetes management appears to help in the reduction of HbA1c levels through interventions that encourage the transmission of patient data, as well as regular and intensive feedback [36]. TM may be more beneficial in people with high HbA1c values, since TM can help patients modify their health behaviors such as diet and physical activity by monitoring them [37].

Metabolic control is a cornerstone in diabetes care and a significant modifier to the risks of diabetes-related complications. Alongside glycemic control, proper management of dyslipidemia, hypertension, and obesity is a well-established protective measure against the development of various micro- and macrovascular complications [38]. While recent decades have witnessed a paradigm shift towards antidiabetic medications with a beneficial impact on overall metabolic control [39], a considerable proportion of patients with T2DM from the MENA region still suffer from poor metabolic control [40]. Our results demonstrated that the TM devices led to significant reductions in blood pressure, body weight, and lipid profile among previously LTFU patients with T2DM. Notably, the reduction in the DBP was clinically relevant with a mean reduction of 3.5 mmHg; the Heart Outcome Prevention Evaluation (HOPE) study found that improvements in systolic and diastolic blood pressure of 3.3 and 1.4 mmHg, respectively, were associated with a 22% reduction in the relative risk of cardiovascular death, myocardial infarction, or stroke [41]. In concordance with our findings, a previous report demonstrated that TM led to a statistically significant reduction in SBP and body mass index (MD = −1.33 mmHg and −0.25 kg/m^2^, respectively) [33]. Similar results were demonstrated in previous reports [35]. However, although HbA1c and number of hypoglycemic events both improved, Lee et al. reported [34] that blood pressure, weight, diabetes distress, and diabetes self-efficacy showed no significant changes with their TM device.

Poor adherence to medication and BG monitoring schedules is a strong independent predictor of inadequate glycemic control in people with T2DM [42]. In this regard, various telehealth modalities were found to improve patients' adherence to antidiabetic medications [43], diabetes self-care [44], and, in return, overall glycemic control [45]. In the present study, we found that the application of the TM approach resulted in adequate patient compliance, as reflected by the high utilization of BG monitoring devices and portable pill dispensers over the study's period. Our findings run in parallel with the current body of evidence highlighting the beneficial role of telemedicine in the patients' adherence to diabetes self-care practices [44].

Despite the reported benefits of telemedicine during the COVID-19 crisis, especially in patients with diabetes, many barriers have been identified, including resistance to change, patient preference for face-to-face visits, concerns about patients' health literacy, and their ability to cope with telemedicine consultations [46]. For patients with diabetes, an additional concern is the potential difficulty of uploading device data independently [47]. The top barriers are technology-specific and could be overcome through training, change-management techniques, and interspersing delivery by telemedicine with personal patient-to-provider interactions [46]. Therefore, it is essential to provide mass awareness campaigns to educate patients with diabetes and answer their concerns regarding the new diabetes management technologies. Offering more telemedicine consultations has the potential to minimize life disruptions, increase engagement opportunities, and allow for the delivery of timely and personalized ongoing diabetes education and training [48].

We acknowledge that this study has some limitations, including the single-center setting and the small sample size, which may hinder the generalizability of our findings. In addition, the causality association between TM implementation and overall diabetes control cannot be confirmed here as the study was a single arm with no control group. The study also did not investigate other risk factors that can interact with the efficacy of TM, including educational level, socioeconomic status, and health literacy.

## 5. Conclusions

TM can serve as an effective tool to support improved glycemic and overall diabetes control in LTFU patients with poor glycemic status. Our single-center experience demonstrated that implementing the TM program, which involved home-based and center-based devices, led to significant improvements in overall diabetes measures, including glycemic control, body weight, and lipid profile. Thus, TM intervention represents an effective solution to engage a challenging population of patients with T2DM who had previously been lost to follow-up, resulting in improvements in metabolic parameters, such as HbA1c, FBG, DBP, weight, total cholesterol, and LDL-cholesterol. Future multicenter studies are required to assess the feasibility and barriers towards the application of a comprehensive TM program in the MENA region.

## Figures and Tables

**Table 1:** Demographic characteristics and medical history of the patients.

Baseline characteristics	Mean	SD
Age	48.2	10.1
	**Count**	**Percent**
Gender		
Male	16	42.1
Female	22	57.9
Medical history		
Any medical history other than diabetes	38	100
Vitamin D deficiency	36	94.7
Dyslipidemia	34	89.5
Obesity	27	71.1
Hypertension	23	60.5
CKD	14	36.8
Microalbumin	9	64.3^*∗*^
Proteinuria	5	35.7^*∗*^
Hypothyroidism	8	21.1
Coronary artery disease	5	13.2
Neuropathy	4	10.5
Prior T2DM medications	**Median**	**IQR**
Number of medications	2.0	3.00
Class of medications	**Count**	**Percent**
Biguanides	23	60.5
DDP4 inhibitors	21	55.3
Insulin secretagogue	15	39.5
SGLT2 inhibitors	11	28.9
Insulin	3	7.9
GLP1	2	5.3
Thiazolidinediones	2	5.3
Current T2DM medications	**Median**	**IQR**
Number of medications	4.0	1.0
Class of medications	**Count**	**Percent**
Biguanides	36	94.7
SGLT2 inhibitors	34	89.5
DDP4 inhibitors	21	55.3
Insulin secretagogue	24	63.2
Insulin	22	57.9
GLP1	18	47.4
Thiazolidinediones	2	5.3
Alpha glucosidase inhibitor	1	2.63

^*∗*^ The percentage was calculated for a total of 14 CKD patients.

**Table 2:** Results of home-based devices of T2DM indicators at baseline and after three months.

Clinical characteristics (mean ± SD)	*N*	Baseline	N	3 months	N with both measures	Difference (95% CI) (3 months–baseline)	*p* value
Blood glucose (mg/dL)							
FBG	26	192.2 (61.4)	31	147.9 (40.3)	20	−40.1 (−70.8–−9.3)	0.013
RBG	32	199.5 (66.4)	25	171.3 (83.3)	21	−40.8 (−84.7–3.2)	0.067
Blood pressure (mmHg)							
SBP	37	135.4 (15.3)	38	133.5 (15.1)	37	−2.2 (−7.2–2.8)	0.386
DBP	37	85.7 (11.7)	38	82.5 (9.4)	37	−3.5 (−6.6–−0.4)	0.028
Pulse rate (beats/min)	37	82.7 (12.5)	38	82.2 (15.4)	37	−0.7 (−4.5–3.1)	0.710
Oxygen saturation (%)	38	97.4 (1.4)	38	95.7 (12.7)	38	−1.8 (−5.9–2.4)	0.393

FBG: fasting blood glucose; RBG: random blood glucose; SBP: systolic blood pressure; DBP: diastolic blood pressure; CI: confidence interval.

**Table 3:** Results of center-based measures of T2DM indicators at baseline and after three months.

Clinical characteristics (mean ± SD)	*N*	Baseline	*N*	3 months	*N* with both measures	Difference (95% CI) 3 months–baseline	*p* value
Bodyweight/Composition (mean ± SD)							
Weight (kg)	37	92.3 (19.8)	37	90.9 (19.7)	36	−1.3 (−2.5–−0.08)	0.037^*∗*^
Fat percent (%)	37	47.7 (10.5)	33	47.7 (9.8)	32	−0.7 (−2.0–0.60)	0.276
Muscle percent (%)	37	48.9 (9.8)	33	48.9 (9.1)	32	0.65 (−0.56–1.85)	0.286
Water percent (%)	37	39.2 (7.4)	33	39.1 (6.9)	32	0.52 (−0.43–1.5)	0.270
Bone weight (kg)	37	3.8 (4.3)	33	3.1 (1.05)	32	−0.82 (−2.5–0.9)	0.331
Spirometry Measurement (mean ± SD)							
FVC (L)	36	2.2 (0.78)	37	2.1 (0.73)	35	−0.04 (−0.28–0.2)	0.768
FEV1 (L)	36	1.8 (0.78)	37	1.8 (0.65)	35	−0.007 (−0.22–0.2)	0.947
PEF (L/S)	36	4.8 (2.5)	37	4.8 (2.5)	35	−0.13 (−0.9–0.6)	0.727
FEV1%	36	84.6 (20.9)	37	86.4 (17.1)	35	1.04 (−8.2–10.3)	0.821
FEF25%	36	4.0 (2.3)	37	4.2 (2.2)	35	0.05 (−0.7–0.8)	0.907
FEF75%	36	1.9 (1.0)	37	1.8 (0.94)	35	−0.11 (−0.3–0.1)	0.313
FEF25-75%	36	3.0 (1.5)	37	2.9 (1.6)	35	−0.09 (−0.5–0.4)	0.683
Blood Tests (mean ± SD)							
FBG (mg/dl)	26	192.2 (61.4)	31	147.9 (40.3)	20	−40.1 (−70.8–−9.3)	0.013^*∗*^
RBG (mg/dl)	32	199.5 (66.4)	25	171.3 (83.3)	21	−40.8 (−84.7–3.2)	0.067
Hemoglobin (g/dl)	35	13.5 (3.0)	37	14.2 (2.0)	34	0.6 (−0.24–1.5)	0.148
Uric acid (mg/dl)	35	6.2 (3.0)	37	6.3 (1.9)	34	0.08 (−0.9–1.04)	0.863
Total cholesterol (mg/dl)	34	154.7 (31.9)	37	134.5 (26.4)	33	−20.6 (−33.9–−7.3)	0.003^*∗*^
HDL-C (mg/dl)	34	41.3 (15.7)	37	43.4 (17.9)	33	2.9 (−4.3–10.2)	0.412
LDL-C (mg/dl)	32	87.5 (26.4)	37	67.2 (22.99)	31	−18.4 (-29.5–−7.3)	0.002^*∗*^
Triglycerides (mg/dl)	34	128.9 (87.0)	37	131 (77.0)	33	−9.0 (−37.3–19.2)	0.520
Serum creatinine (mg/dl)	35	0.99 (0.4)	37	1.17 (0.26)	34	0.16 (−0.005–0.300)	0.056
Urine Tests (mean ± SD)							
Glucose (*μ*mol/L)	37	33.6 (25.6)	38	41.05 (23.8)	37	7.0 (−2.2–16.3)	0.131
Bilirubin (*μ*mol/L)	37	10.0 (15.9)	38	11.6 (17.3)	37	1.9 (−5.9–9.6)	0.628
Specific gravity	37	1.02 (0.008)	38	1.02 (0.009)	37	0. (−0.008–0.0)	0.027^*∗*^
Ketones (*μ*mol/L)	37	0.04 (0.25)	38	0	37	−0.04 (−0.12–0.04)	0.324
Occult blood (*μ*mol/L)	37	0	38	6.58 (40.6)	37	6.7 (−6.9–20.5)	0.324
Proteins (*μ*mol/L)	37	0.34 (0.70)	38	0.34 (0.62)	37	0.007 (−0.25–0.26)	0.955
Urobilinogen (*μ*mol/L)	37	3.3 (0)	38	3.2 (0.54)	37	−0.09 (−0.27–0.09)	0.324
Nitrites (*μ*mol/L)	37	0.49 (2.96)	38	0	37	−0.486 (−1.5–0.50)	0.324
Leukocytes (cells/*μ*l)	37	13.9 (82.2)	38	0	37	−13.9 (−41.3–13.5)	0.310
Vitamin C (*μ*mol/L)	37	0.016 (0.10)	38	0.2 (0.93)	37	0.19 (−0.13–0.5)	0.235
Ph	37	5.95 (0.23)	38	5.89 (0.4)	37	−0.05 (−0.19–0.08)	0.422
ECG measurement (%Normal)							—
P wave	37	100	37	100		—	
PR interval	37	100	37	100		—	
QRS complex	37	100	37	100		—	
ST segment	37	100	37	100		—	
T wave	37	100	37	100		—	
QT interval	37	100	37	100		—	

**Table 4:** Mean number of days the participants used home-based devices and required number of reminders needed.

Home-based devices	Mean number of days used	SD	Median	IQR
OneTouch® select plus Flex® BGM	72.9	23.5	79	40
Electronic sphygmomanometer	62.3	28.6	71	49
Pulse oximeter	50.4	28.6	47	39
Portable pill dispenser	86.5	22.8	91	19
Number of reminders per patient	2952	935.5	2837	1306

^*∗*^ SD: standard deviation.

## Data Availability

The data used to support the findings of this study are included within the article.

## Abbreviations

BG: Blood glucose
CI: Confidence interval
COVID-19: Coronavirus disease 2019
DDC: Dubai Diabetes Center
ECG: Electrocardiography
FBG: Fasting blood glucose
IRB: Institutional Review Board
LTFU: Loss-to-follow-up
MD: Mean difference
MENA: Middle East and North Africa
RBG: Random blood glucose
T2DM: Type 2 diabetes mellitus
TC: Teleconsultations
TM: Telemonitoring
UAE: United Arab Emirates.

## Supplementary Materials

Supplementary Materials Appendix 1. Classes of Medications taken by each patient at the end of the study.
