# Supplementary material for: The Impact of Telemonitoring on Improving Glycemic and Metabolic Control in Previously Lost-to-Follow-Up Patients with Type 2 Diabetes Mellitus: A Single-Center Interventional Study in the United Arab Emirates
Source: Int J Clin Pract. 2022 Apr 20;2022:6286574. doi: 10.1155/2022/6286574 (PMC9159213; doi:10.1155/2022/6286574)
Supplement: Supplementary Materials — Appendix 1. Classes of Medications taken by each patient at the end of the study. [file 6286574.f1.pdf]

**Appendix 1. Classes of Medications taken by each patient at the end of the study**

| | | Diabetic Medications Class | | | | | | | |
|---|---|---|---|---|---|---|---|---|---|
| | | Insulin | SGLT2 | Biguanides | DPP4 | Insulin secretagogue | sulfonylurea | GLP1 | Thiazolidinediones |
| Patient No | 1.00 | 0 | X | X | X | X | 0 | 0 | 0 |
| | 2.00 | 0 | X | X | X | 0 | X | 0 | 0 |
| | 3.00 | 0 | X | X | X | X | 0 | 0 | 0 |
| | 4.00 | 0 | X | X | 0 | X | 0 | 0 | 0 |
| | 5.00 | 0 | X | X | X | X | 0 | 0 | X |
| | 6.00 | 0 | 0 | X | X | 0 | 0 | 0 | 0 |
| | 7.00 | 0 | X | X | X | X | 0 | 0 | X |
| | 8.00 | 0 | 0 | X | X | X | 0 | 0 | 0 |
| | 9.00 | 0 | X | 0 | X | 0 | 0 | 0 | 0 |
| | 10.00 | X | X | X | X | X | 0 | X | 0 |
| | 11.00 | 0 | X | X | X | X | 0 | X | 0 |
| | 12.00 | 0 | X | X | X | 0 | 0 | 0 | 0 |
| | 13.00 | 0 | X | 0 | 0 | X | 0 | 0 | 0 |
| | 14.00 | X | X | X | 0 | 0 | 0 | X | 0 |
| | 15.00 | 0 | 0 | X | 0 | X | 0 | 0 | 0 |
| | 16.00 | 0 | X | X | X | X | 0 | 0 | 0 |
| | 17.00 | 0 | 0 | X | X | 0 | 0 | X | 0 |
| | 18.00 | X | 0 | X | X | 0 | 0 | X | 0 |
| | 19.00 | X | X | X | 0 | X | X | 0 | 0 |

| | | | | | | | | |
|---|---|---|---|---|---|---|---|---|
| 20.00 | X | X | X | 0 | 0 | 0 | X | 0 |
| 21.00 | 0 | X | X | X | 0 | X | 0 | 0 |
| 22.00 | 0 | X | X | X | 0 | 0 | 0 | 0 |
| 23.00 | 0 | X | X | X | X | 0 | 0 | 0 |
| 24.00 | 0 | X | X | X | 0 | 0 | 0 | 0 |
| 25.00 | 0 | 0 | X | X | X | 0 | 0 | 0 |
| 26.00 | X | X | X | X | X | 0 | 0 | 0 |
| 27.00 | X | X | X | X | 0 | 0 | X | 0 |
| 28.00 | 0 | X | X | X | X | 0 | 0 | 0 |
| 29.00 | 0 | X | X | X | 0 | X | 0 | 0 |
| 30.00 | 0 | X | X | X | X | 0 | 0 | 0 |
| 31.00 | 0 | X | 0 | X | X | 0 | X | 0 |
| 32.00 | 0 | X | 0 | X | 0 | 0 | 0 | 0 |
| 33.00 | 0 | 0 | X | X | 0 | 0 | 0 | 0 |
| 34.00 | X | 0 | X | 0 | X | 0 | X | 0 |
| 35.00 | X | X | X | 0 | X | 0 | X | 0 |
| 36.00 | 0 | X | X | 0 | X | X | 0 | 0 |
| 37.00 | X | X | X | 0 | X | 0 | X | 0 |
| 38.00 | X | X | X | X | 0 | 0 | X | X |

**X: Patient taking the medication**

**0: Patient not taking the medication**
